# Thermal Processing Techniques Differentially Modulate Phytochemicals, Antioxidant Potential, and Genoprotective Effects of Kale (*Brassica oleracea* var. *acephala*) and Chard (*Beta vulgaris* L. var. *cycla*)

**DOI:** 10.3390/plants14243808

**Published:** 2025-12-14

**Authors:** Marta Frlin, Karlo Miškec, Ivana Šola

**Affiliations:** Department of Biology, Faculty of Science, University of Zagreb, Horvatovac 102a, 10000 Zagreb, Croatia; marta.frlin@biol.pmf.unizg.hr (M.F.);

**Keywords:** air-frying, DNA nicking protection assay, hierarchical clustering, phenolics, principal component analysis

## Abstract

Thermal processing alters the nutritional and functional properties of vegetable food. In this study, using electrophoretic, spectrophotometric, and statistical analyses, we analyzed the effects of boiling, blanching, steaming, and blanching followed by pan-frying and air-frying on the concentration of bioactive compounds in kale and chard, and the biological effects of their extracts. In addition to analyzing the vegetable tissues, the residual water remaining after thermal processing was also examined to assess the nutritional potential of this often overlooked and typically discarded by-product. The residual cooking water had the highest antioxidant capacity, according to ABTS, DPPH, and FRAP assays (57.83% ± 18.16%, 33.58% ± 16.55%, and 81.58% ± 0.78% for kale and 74.53% ± 4.56%, 13.62% ± 7.34%, and 82.97% ± 0.44% for chard, respectively). Air-frying and cooking water contained the highest total phenolics (0.48 ± 0.17 mg GAE/g fw and 0.35 ± 0.06 mg GAE/g fw for kale and 0.88 ± 0.21 mg GAE/g fw and 0.80 ± 0.06 mg GAE/g fw for chard, respectively). Thermally processed chard had a higher concentration of oxidative marker H_2_O_2_ than kale. An inverse relationship between soluble sugars and H_2_O_2_ levels was observed. In kale, cooking processes caused the greatest reduction in soluble sugars, whereas in chard, this effect was most pronounced during blanching. Chard had more photosynthetic pigments than kale. Heat treatments caused more differences between kale samples than chard samples. Pan-frying best preserved chlorophylls, porphyrins, and carotenoids. According to both PC and HC analysis, the tissues of kale were clearly distinguishable from the water remaining after boiling/blanching/steaming kale. These results may help to optimize industrial processing conditions to better preserve bioactive compounds and create opportunities for the valorization of cooking by-products.

## 1. Introduction

In modern society, poor dietary habits are a major contributor to the risk of chronic diseases. Non-communicable diseases such as cardiovascular disease, cancer, diabetes, and chronic respiratory conditions account for the majority of global deaths [1]. Substantial evidence supports the encouragement of ingesting different vegetable groups that prevent chronic diseases [2,3]. Vegetables are rich in different antioxidant molecules (mostly polyphenolics), as well as fibers and vitamins, which have an anti-inflammatory effect on the body [4]. These effects have been proven to improve cardiovascular health, neurodegenerative disorders and ocular disorders [5]. Kale (*Brassica oleracea* var. *acephala*) from the family Brassicaceae and chard (*Beta vulgaris* L. var *cycla*) from the Amaranthaceae family contain high levels of phenolic compounds and carotenoids, with kale also containing extremely bioactive glucosinolates [6]. Kale, which originates from eastern Turkey, is one of the oldest cultivated cruciferous vegetables and has been used in culinary practices for centuries due to its affordability and resilience to environmental stress [7]. It was usually used to treat gastric ulcers, high cholesterol, rheumatism, and hepatic diseases [8,9]. It is mostly high in indolic and aliphatic glucosinolates, as well as glycosides of the flavonoids quercetin, kaempferol, and isorhamnetin [10,11,12]. On the other hand, chard originates from the Mediterranean region, specifically Sicily. It was shown that it can reduce blood sugar and improve liver state in diabetic rats [13]. Chard was used before main phytochemicals were known to humans; however, today we know that it is rich in fatty acids [13], phospholipids and glycolipids [14], and ascorbic and folic acid [15], as well as saponins and flavonoids [13] that mitigate the hypoglycemic effect.

The major downside of the consumption of raw vegetables is their hardiness and lack of flavor. Another downside is low bioaccessibility of primary and/or specialized metabolites from some raw vegetables. These can be affected by developmental stages of the plant, harvest season, variety, and preparation techniques [16]. For example, treatment with heat increased the bioaccessibility of cardoon (*Cynara cardunculus* L.) polyphenols from 2% of raw cardoon to even 60–67% of cooked cardoon [17]. One of the possible reasons is the fact that the cooking process loosens the cell wall structure and, that way, enables easier extraction of polyphenolics [18]. There are several different cooking methods used today for vegetables, with boiling, blanching, steaming, pan-frying, and air-frying being the most used methods [19]. Cruciferous vegetables are usually boiled or steamed for 5 min in or above a pot with boiling water [20,21]. It was shown that, due to the extraction of chemicals into water, the concentration of phytochemicals like polyphenols and flavonoids in sathkora (*Citrus macroptera*), pumpkin (*Cucurbita maxima*), green peas (*Pisum sativum*), pepper (*Capsicum annuum*), and spinach (*Spinacia oleracea*) decreased after boiling and steaming [22]. Change in the concentration of phytochemicals also depends on the vegetable type. For example, the concentration of isothiocyanates increased in boiled cauliflower (*B. oleracea* var. *botrytis*) by 1.8-fold, whereas it decreased in kale by 0.8-fold, compared to raw [20]. In mature broccoli (*B. oleracea* var. *italica*), a great loss of chlorophyll, vitamin C, and glucosinolates [21], as well as total phenols, flavonoids, hydroxycinnamic acids, tannins, and proanthocyanidins [19] was detected while boiling compared to steaming. Blanching vegetables is usually conducted for 30–180 s in boiling water [23]. When kale is blanched, it results in the retention of 86.9%, 55.6%, 27.6%, and 12.9% of vitamin B1, B3, C, and potassium, respectively, compared to raw [24]. However, blanched white cauliflower has stronger antioxidant potential and a higher concentration of protocatechuic acid, quercetin, pyrogallol, vanillic acid, and kaempferol compared to boiled [25]. Pan-frying is usually performed for 2–3 min in vegetable to oil, in the ratio of 10:1, and has been shown to be the least invasive method of cooking for maximum bioavailability and nutrient content [21,26]. For example, it was shown that stir-frying did not reduce the total glucosinolates and vitamin C content of broccoli compared to raw [26]. Air-frying is usually conducted for 10 min at 160 °C [27]. Although air-frying can negatively affect the ability to scavenge ABTS radicals in some vegetables, like colored bell peppers (*C. annuum*) [28,29], it is a better method for mature broccoli [19], canola, mustard, kale, broccoli sprouts, red cabbage, and green cabbage than boiling [27]. Since thermal processing can affect the polyphenol content and antioxidant capacity of vegetables, selecting the least invasive preparation method is crucial to preserve their nutritional value. Moreover, because heat inactivates enzymes that contribute to quality degradation, it is important to tailor thermal processing techniques for each vegetable type to minimize undesirable nutrient losses [23].

This study aimed to evaluate the impact of five thermal processing techniques—boiling, blanching, steaming, pan-frying, and air-frying—on the phytochemical composition, antioxidant capacity, and DNA-protective effects of kale (*B. oleracea* var. *acephala*) and chard (*B. vulgaris* subsp. L. var *cycla*). Since different cooking techniques specifically affect nutrient retention in thermally processed vegetables, we tried to find the most adequate for preserving nutritional value in kale and chard, as well as in residual cooking water (referred also as aqueous extract), where applicable. For the first time, we also investigated the genoprotective effects of thermally processed kale and chard on the plasmid DNA structure, using the densitometry of DNA exposed to an external ROS source. We further applied principal component analysis (PCA) and hierarchical clustering (HC) to evaluate the overall effects of each processing method. In our previous study [19], we showed that steaming and air-frying best preserved the nutritional value of broccoli. In the present study, we aimed to determine whether a single cooking method could maximize the nutritional value of both kale and chard, or whether the optimal method differs, depending on the vegetable type.

## 2. Results and Discussion

### 2.1. Genoprotective Effects of Kale and Chard Subjected to Different Thermal Processing Techniques

**Figure 1:**
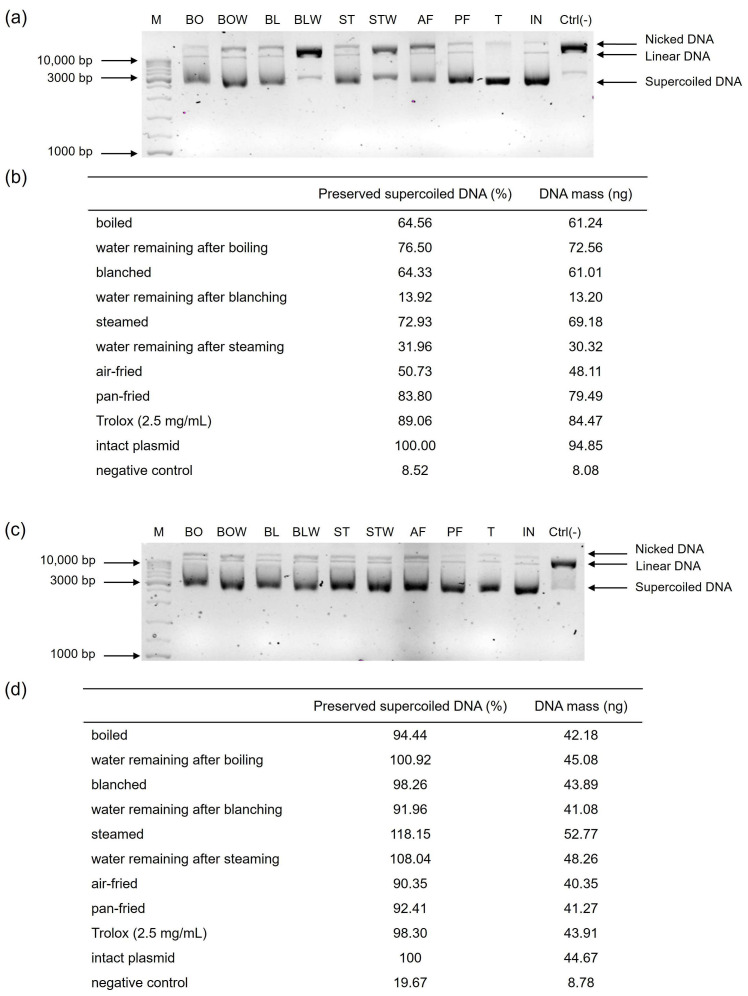
Effect of thermal processing on the potential of (**a**,**b**) kale and (**c**,**d**) chard extracts to protect plasmid supercoiled DNA from damage caused by Fenton’s reagent. The results are expressed relative to the concentration of the supercoiled DNA of an intact plasmid. (**a**,**c**) The 1.5% agarose gel with three distinct plasmid conformations (supercoiled, linear, and nicked). (**b**,**d**) The percentage of preserved supercoiled DNA conformation after exposure to the Fenton’s reagent relative to the intact plasmid, alongside the corresponding DNA mass calculated from the standard curve, expressed in nanograms. A band densitometry was performed using the ImageJ (Fiji) software, version 2.2.0. M = GeneRuler DNA Ladder Mix (Thermo Fisher Scientific, Waltham, MA, USA); BO = boiled; BOW = water remaining after boiling; BL = blanched; BLW = water remaining after blanching; ST = steamed; STW = water remaining after steaming; AF = air-fried; PF = blanched and then pan-fried; T = Trolox 2.5 mg/mL; IN = intact plasmid; Ctrl(-) = negative control; bp = base pair.

Plants contain antioxidant molecules that show, to a certain degree, a genoprotective effect on DNA molecules [30]. An in vitro DNA nicking protection assay was performed to evaluate the effects of differently thermally processed kale and chard on the supercoiled form of plasmid DNA. Extracts from the processed vegetables exhibited varying capacities to protect plasmid DNA from oxidative stress-induced nicking (Figure 1). Pan-fried kale was the most effective in preserving the supercoiled DNA form (83.80%), which was comparable to 2.5 mg/mL solution of Trolox. On the other hand, water that remained after blanching kale had the lowest ability to preserve the supercoiled plasmid DNA structure (13.92%), which could be associated with the lowest concentration of phytochemicals. Because kale has a robust cell wall, brief steaming may have been insufficient to release antioxidant molecules, resulting in a lower DNA-protective effect. Since polyphenolics are hydrophilic, they leach out during higher temperatures, which contributes to the genoprotective effects, as stated in Miškec et al., 2025 [19]. However, that was not the case in our study, at least for kale, while in aqueous chard extracts, genoprotective effects were detected. Water that remained after boiling kale showed higher genoprotective effects (76.50%) compared to tissue (64.56%), probably due to the extraction of phytochemicals. 

Since this is the first time investigating the genoprotective effects of kale and chard extract, there are no examples from the literature to compare our results with. Research on the genoprotective effects of thermally processed plant extracts remains limited, largely because the DNA nicking protection assay is a specialized and labor-intensive method compared with conventional antioxidant assays. This technique requires the handling and amplification of plasmid DNA, strict control of reaction conditions, and meticulous optimization, making it uncommon in mainstream phytochemistry or thermal-processing research. Its low throughput and substantial time demand further discourage its use when processing large numbers of samples. Moreover, DNA protection assays fall at the interface between food science and biomedical research, contributing to their underrepresentation in studies that are focused primarily on phytochemical changes. The need for dedicated molecular biology equipment and familiarity with genotoxicity testing also restricts its application in many phytochemistry laboratories. In addition, reproducibility can be challenging, as each assay must be carefully standardized with respect to DNA type and concentration, levels of oxidative agents, incubation conditions, and plasmid extraction methods. Finally, DNA-protective outcomes are more difficult to interpret and relate directly to processing parameters than traditional chemical antioxidant measurements, further limiting the routine use of this assay. Although air-fried samples showed a higher concentration of genoprotective chemicals (total phenols, proanthocyanidins, and flavonols, as shown in Table 1), DNA could still be oxidized and degraded to its nicked and linear form [31], due to high H_2_O_2_ levels (Table 2). It is possible that H_2_O_2_ was extracted from the tissue and produced hydroxyl radicals to damage the DNA.

**Figure 2:**
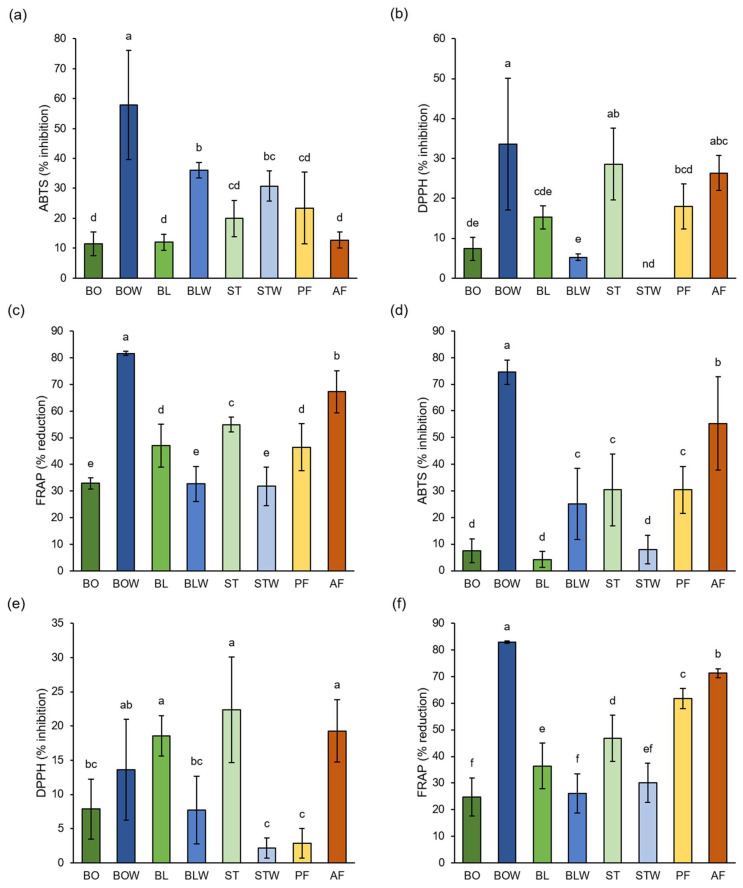
Effect of different thermal processing techniques on antioxidant capacity of (**a**–**c**) kale and (**d**–**f**) chard, determined by (**a**,**d**) ABTS method, (**b**,**e**) DPPH method, and (**c**,**f**) FRAP method. Values represent average ± standard deviation of three biological and four technical replicates. Different letters indicate a significant difference among the values (one-way ANOVA, Duncan’s test, *p* ≤ 0.05). BO = boiled; BOW = water remaining after boiling; BL = blanched; BLW = water remaining after blanching; ST = steamed; STW = water remaining after steaming; PF = blanched and then pan-fried; and AF = air-fried.

Thermally processed chard samples were more effective in preserving the supercoiled DNA form than kale, with steamed chard being the most effective (118.15%), and air-fried chard the least effective (90.35%). This could be attributed to the reduced leaching and oxidative degradation of antioxidants during steaming compared to air-frying, which exposes samples to higher temperatures and more oxidative conditions. The DNA-protective potential of all chard samples was comparable with Trolox 2.5 mg/mL. This corresponds with the higher total phenol content observed in pan-fried chard and the elevated total tannin levels in steamed chard (Table 1b), as well as their respective antioxidant capacities—DPPH for steamed chard and FRAP for pan-fried chard (Figure 2b). According to [19], polyphenols form strong complexes with iron which lower its concentration in Fenton’s reagent and reduce the formation of hydroxyl radicals. Water remaining after boiling chard had a higher genoprotective activity than corresponding tissue, while water remaining after blanching and steaming were less effective in preserving the supercoiled DNA form than their corresponding tissues. A longer cooking time for boiling (30 min, compared to 30 s for blanching and 5 min for steaming) and direct contact with water resulted in the extraction of a greater amount of polyphenols (Table 1b) responsible for its higher DNA-protective activity. Thermal processing not only alters the chemical composition of vegetables but also induces substantial changes in their microstructure, which directly influence the behavior of bioactive compounds. Heat treatments disrupt cell walls, soften pectic substances, rupture membranes, and modify the integrity of tissues such as the epidermis, mesophyll, and vascular bundles. These structural transformations alter the release, stability, and mobility of phytochemicals, determining whether compounds are retained within the tissue or leach into the cooking water. Vegetables with softer tissues, such as chard, generally exhibit greater cell-wall breakdown and compound release than those with thicker cuticles or more rigid tissues, such as kale. Because the DNA-protective capacity depends on the accessibility and concentration of antioxidants and phenolic constituents, the microstructural effects of each thermal process play a key role in shaping the genoprotective outcomes observed. Including microstructural considerations therefore provides a more mechanistic understanding of how thermal treatments modulate phytochemical availability and DNA protection.

### 2.2. Influence of Different Thermal Processing Techniques on the Antioxidant Potential of Kale and Chard

Thermal processing notably affected the antioxidant capacity of kale and chard, as determined by ABTS, DPPH, and FRAP assay (Figure 2). The highest antioxidant potential of kale extracts was recorded in the water remaining after boiling, according to all three methods (57.83% ± 18.16%, 33.58% ± 16.55% and 81.58% ± 0.78%, respectively). DPPH results also indicate that steamed and air-fried kale have a high antioxidant potential (28.58% ± 8.97% and 26.37% ± 4.32%, respectively). The water remaining after boiling kale contained the highest levels of total tannins, flavonoids, and hydroxycinnamic acids (Table 1a), suggesting that these compounds were primarily responsible for its strong antioxidant potential. Air-fried kale had the highest amount of total phenolics, proanthocyanidins, and flavonols (Table 1a). Our results show no significant difference between pan-fried and steamed kale, according to the ABTS and DPPH assays, whereas steamed kale had a higher antioxidant potential than boiled one, according to the DPPH and FRAP. According to Akdaş et al. [32], stir-fried kale had lower antioxidant potential than steamed kale, measured by the DPPH method, while no significant difference was recorded between boiled and steamed kale, according to the ABTS and DPPH methods. In their study, boiling, steaming, and stir-frying were conducted for 5 min, while in our study, boiling lasted for 30 min, steaming for 3 min and pan-frying for 5 min. Another study reported that the highest antioxidant potential measured by ABTS was in the boiled kale; it was lower in steamed and the lowest in stir-fried kale [33]. In this study, boiling and stir-frying were performed for 4 min and steaming for 5 min. The FRAP results indicated that air-fried kale exhibited higher antioxidant potential than steamed kale, whereas the ABTS and DPPH assays showed no significant differences. These findings are consistent with those reported by Nandasiri et al. [27], who also recorded higher antioxidant potential of air-fried kale than steamed kale, measured by FRAP. However, they reported opposite results when the antioxidant potential was assessed using DPPH. In our study, all three assays indicated that the water remaining after boiling exhibited higher antioxidant potential than the corresponding vegetable tissue. However, according to the DPPH and FRAP assays, water that remained after blanching and steaming had a lower antioxidant potential than associated tissue.

Studies using these three methods have shown that different thermal processing techniques produce varying antioxidant potentials in broccoli [19,34,35]. Since FRAP detects hydrophilic antioxidants, it is possible that in our experiments, water that remained after boiling had the highest antioxidant capacity due to retention of soluble antioxidant radicals, while DPPH usually detects lipophilic antioxidants [35,36].

Results from all three methods (ABTS, DPPH, and FRAP) also show the highest antioxidant potential as being in the water remaining after boiling chard (74.53% ± 4.56%, 13.62% ± 7.34%, and 82.97% ± 0.44%, respectively). DPPH results showed that blanched, steamed, and air-fried chard had the highest antioxidant capacity (18.56% ± 2.96%, 22.35% ± 7.72%, and 19.30% ± 4.57%, respectively). Both ABTS and FRAP also suggest a relatively high antioxidant capacity for air-fried chard. Water that remained after boiling chard had the highest amount of all measured phenolics (Table 1b), which are known as acting antioxidants and contribute to its antioxidant potential. Air-fried chard also had a high amount of total phenolics, tannins, flavonoids, and flavonols (Table 1b). Data on the effects of different thermal processing techniques on the antioxidant potential of chard are limited, and most existing studies report changes that are relative to the raw vegetable without comparing the effects of different processing methods. A previous study on chard showed that boiling did not result in the loss of antioxidant potential, measured by ABTS, compared to raw chard, while frying lowered the antioxidant potential [37]. In their study, boiling was conducted for 20 min and frying for 10 min (30 min and 5 min in our study, respectively), which could explain why our results suggest a higher antioxidant potential of pan-fried than boiled chard. Due to the longer cooking time, most antioxidants were extracted or degraded during boiling, whereas the shorter cooking time preserved more antioxidants while pan-frying. Another study [38] also suggests that boiling chard for 8 min did not change its antioxidant potential, measured by ABTS and DPPH. In this study, the ABTS and FRAP results show that water remaining after boiling had a higher antioxidant potential than the associated tissue, while DPPH and FRAP showed that the water that remained after blanching had a lower antioxidant capacity than the associated tissue. All methods indicate that steamed chard had higher antioxidant potential than the water that remained after thermal processing. Boiling was performed for a much longer duration than blanching or steaming (30 min, 30 s, and 3 min, respectively), allowing for more antioxidants to leach from the tissue into the boiling water, whereas most were retained during blanching and steaming. All methods (except ABTS for blanched chard) showed that blanched and steamed chard exhibited higher antioxidant potential than boiled chard, which was expected due to their shorter cooking times. We previously recorded the same relationship between tissue and the remaining water of broccoli [19]. While heat treatments can degrade some phenolic compounds, resulting in potential losses of antioxidant capacity, other factors can contribute to high observed antioxidant values. One important factor is the formation of Maillard reaction products (MRPs) during heating, especially in the presence of amino acids and reducing sugars. These MRPs are known to exhibit antioxidant activity, which can partially compensate for the loss of native phenolics [39]. Additionally, heat can sometimes increase the extractability of certain bound phenolic compounds, making them more measurable [40]. Therefore, the high antioxidant capacity observed in some heat-treated samples may result from a combination of residual phenolics, enhanced extractability, and the formation of Maillard reaction products.

### 2.3. Influence of Different Heat Treatment Techniques on Specialized Metabolites in Kale and Chard

Phenolic compounds contribute significantly to the antioxidant potential of plant extracts [41]. Different thermal processing techniques of kale resulted in differential polyphenolic compositions of aqueous and ethanolic extracts (Table 1a). The highest amount of total phenolics was found in air-fried kale (0.48 ± 0.17 mg GAE/g fw) and cooked (0.43 ± 0.24 mg GAE/g fw) kale, followed by all aqueous extracts. Contrary to our result, it was previously reported that boiled kale had a lower amount of total phenolics than steamed and stir-fried kale [33]. In agreement with our results, Nandasiri et al. reported that air-frying kale resulted in a higher level of total phenolics than steaming. However, they recorded a higher level of total flavonoids after air-frying as well [27]. Similar results were shown on broccoli, in which the highest amount of total phenols was found after air-frying [19]. However, cooked broccoli had fewer total phenols than blanched or steamed, due to the extraction of phytochemicals into the water, where they showed a higher concentration. Kale contains higher levels of phenolic acids, such as ferulic and caffeic acids, whereas broccoli is richer in flavonols, including quercetin and kaempferol, and possesses a greater proportion of water-soluble and heat-labile phenolic compounds [42,43,44]. Cooked kale contains a larger initial pool of total phenols compared to broccoli, so it is not surprising that its total phenol content is higher after cooking. Different phenolic compounds have varying solubility properties; however, heat generally enhances their solubility in water [45]. Therefore, a relatively high amount of total phenols can also be found in water after cooking (0.35 ± 0.06 mg GAE/g fw), blanching (0.31 ± 0.13 mg GAE/g fw), and steaming (0.39 ± 0.28 mg GAE/g fw) kale. Similarly, it was shown that microwaving four *Boletus* mushrooms better preserved the phenolics than cooking, due to the extraction of chemicals into the water [46]. Potentially, there could be a relationship between Folin–Ciocâlteu reagent and melanoidins in the total phenol analysis. However, our concentrations of total phenols are similar to other authors for raw and thermally processed kale [33]. Thermal processing typically causes cell damage and disrupts polysaccharide fibers [47], leading to molecular oxidation and a reduction in total phenolics. However, this was not observed in our study, likely due to the robust nature of kale’s cell membranes. Although steaming is generally considered to be a gentler method than boiling because the vegetables are not immersed in water, the hardy structure of kale may make its antioxidants more susceptible to oxidation by hot steam than by boiling. Contrary to our results, broccoli sprouts and kale had a higher amount of total phenols after air-frying [27] compared to boiled or steamed. Water remaining after boiling kale contained the highest concentration of total tannins (0.25 ± 0.09 mg GAE/g fw), flavonoids (0.99 ± 0.28 mg QE/g fw), and hydroxycinnamic acids (2.20 ± 0.36 mg CAE/g fw). Flavonoids, as well as tannins, are also somewhat water-soluble [48]. During boiling, cell walls soften and rupture, which allows for these compounds to be extracted into the water [49]. Additionally, heat disrupts vacuolar membranes and the cellulose-pectin wall, which trap specialized metabolites [50]. Tannins are also moderately resistant to heat, which leaves them intact in the water after cooking [51]. Similar results were shown in broccoli, for which the highest amount of flavonoids was recorded in the water after boiling compared to other cooking methods [19]. Air-fried kale had the highest level of total phenolics, proanthocyanidins, and flavonols (0.48 ± 0.17 mg GAE/g fw, 0.15 ± 0.05 mg CatE/g fw and 0.50 ± 0.04 mg QE/g fw, respectively). Air-frying is generally considered to be the most sustainable thermal processing method for enhancing bioactive compounds in *Brassica* vegetables, due to its shorter cooking time, minimal water contact, and the formation of new antioxidant molecules through the Maillard reaction [27,39]. Similar results were shown on broccoli, in which air-frying resulted in the highest amount of total hydroxycinnamic acids and proanthocyanidins compared to boiling, steaming, blanching, and pan-frying [19]. Pan-frying is generally regarded as a less favorable cooking method because the higher temperatures achieved compared to boiling or air-frying increase membrane permeability and promote the degradation of bioactive compounds. It also extracts the lipophilic contents and depletes their nutrient content [52]. However, it was shown that hydroxycinnamic acids from red onion skin had better availability when being pan fried in comparison to baking and grilling [53]. Similar results were shown on potatoes, in which pan-fried samples showed a higher amount of hydroxycinnamic acids compared to other cooking methods [54]. Air-frying generally has mild dehydration and shorter heating time, which limits the extraction of phytochemicals and makes them concentrated in the tissue [27]. Moderate heat breaks the walls and releases bound chemicals [55], while some phenolics form complexes with Maillard intermediates, improving their antioxidant stability [27,39]. Since air-frying is a low-oil and low-water cooking method, more phytochemicals are conserved. A substantial extraction of polyphenols into the cooking water was observed for kale (0.35 ± 0.06 mg GAE/g fw, 0.31 ± 0.13 mg GAE/g fw, and 0.39 ± 0.28 mg GAE/g fw for boiling, blanching, and steaming, respectively). All flavonols and hydroxycinnamic acids were more likely to be retained in the tissue during blanching compared to other cooking methods. The tissue and residual water after steaming exhibited similar levels of various polyphenols. Some studies suggest that hydroxycinnamic acids and flavonols are preferentially retained in the tissue, rather than being lost to the water during steaming. However, different results have been reported for cooked broccoli. The hydroxycinnamic acid content was lower in tissue compared to water extracts, which could be due to the length of the cooking process [56].

Air-fried chard and water remaining after cooking had the highest amount of total phenols (0.88 ± 0.21 mg GAE/g fw, 0.80 ± 0.06 mg GAE/g fw, respectively), tannins (0.37 ± 0.17 mg GAE/g fw and 0.43 ± 0.17 mg GAE/g fw, respectively), flavonoids (1.86 ± 0.14 mg QE/g fw and 1.74 ± 0.28 mg QE/g fw, respectively), and flavonols (1.49 ± 0.60 mg QE/g fw and 1.57 ± 0.07 mg QE/g fw, respectively) (Table 1b). Similar results were found for air-fried eggplants [57]. This is due to air-frying being the least invasive cooking method, while water after cooking extracts most of the hydrophilic phytochemicals. Similar results were shown for broccoli as well [19]. The highest amount of tannins in tissue extracts was preserved in pan-fried chard (0.18 ± 0.07 mg GAE/g fw) and air-fried chard (0.37 ± 0.17 mg GAE/g fw), due to pan-frying releasing tannins due to the aggressive disruption of cell walls and lack of water to leach, while in water extracts, most of the tannins leached and show a higher amount due to their solubility [58]. However, the highest amount of tannins was conserved in water after boiling chard (0.43 ± 0.17 mg GAE/g fw). Both broccoli [19] and chard had a high amount of tannins in the air-fried and pan-fried samples. Regarding steaming, this method uses less water than boiling; however, condensed steam collects on the leaf surface and dissolves tannins, which then leach into the water. As a result, steamed samples exhibit a lower tannin content compared to those processed by air-frying. Water that remained after cooking contained the highest level of proanthocyanidins as well (0.46 ± 0.22 mg CatE/g fw) and hydroxycinnamic acids (7.83 ± 0.35 mg CAE/g fw). The high level of proanthocyanidins is probably due to their water solubility. Most monomers, such as catechin and epicatechin, are water soluble, whereas oligomers and polymers are poorly soluble in water, which could explain the presence of proanthocyanidins in both water and tissue, depending on the type [59]. A substantial extraction of polyphenols into water was also observed for chard. After boiling chard, all groups of polyphenolics were more abundant in the remaining water after thermal processing than in boiled tissue. On the other hand, most polyphenolic groups were retained in thermally processed tissue after blanching, since blanching does not involve longer exposure to hot water (30 min for boiling, compared to 30 s for blanching); therefore, this is a less disruptive thermal process than cooking. The tissue and water remaining after steaming had similar levels of different polyphenolic groups, probably due to not having direct contact with water. The ratio of polyphenols between tissue and water remaining after cooking corresponds to their genoprotective activities (Figure 1). There is limited research on the influence of thermal processing of polyphenolic content of chard. A previous study reported that boiling reduced the levels of total phenolics compared to raw, while pressure-cooking and microwaving did not [60]. The bioactive compound content measured in the fried kale and chard reflects only the fraction remaining in the plant material after frying. During the frying process, a portion of these compounds can leach into the oil, and some may also degrade due to heat. Measuring the compounds in the frying oil provides complementary information, representing the fraction lost from the vegetables. By considering both the fried vegetables and the oil, a more accurate estimate of the original bioactive compound levels in kale and chard can be obtained, although some heat-induced degradation may result in a slightly lower total than in the raw plants. Therefore, the compound amounts in the oil partially confirm the composition of the vegetables and help account for losses during frying. However, we did not measure the composition in oil.

Different thermal processing techniques did not result in significantly different levels of the total intact glucosinolates in kale and chard (Table 1). Regardless of the cooking method used, all samples exhibited similar glucosinolate levels. Since glucosinolates are thermally stable in the absence of myrosinase [61], it is likely that the duration of our cooking treatments was too short to cause significant degradation, leaving these compounds largely intact and protected within the cell walls and membranes. Especially in air- or pan-frying, although temperatures are high, the leaf interior could protect glucosinolates, since kale and chard have a hardy membrane. It is also known that blanching and boiling deactivate myrosinase and without its enzymatic activity, glucosinolates remain intact [62]. Although our results did not show significant changes in glucosinolate content in kale and chard after thermal processing, Korus et al. [63] reported that kale experienced a substantial loss of total glucosinolates—42% after boiling and 30% after blanching. Additionally, mostly, indole glucosinolates have a higher sensitivity to cooking than aliphatic glucosinolates [63]. Kale is rich in aliphatic glucosinolates, including glucoerucin, glucoraphanin, progoitrin, gluconapin, glucoiberin, and glucobrassicanapin [64]; however, no information regarding glucosinolate content was found for chard.

### 2.4. Changes in Soluble Sugar Content and Hydrogen Peroxide of Kale and Chard Under Different Thermal Processes

Air-fried kale had the highest amount of soluble sugars (15.30 ± 0.83 mg SucE/g fw) and the amount was relatively high in steamed kale (10.95 ± 4.71 mg SucE/g fw) as well (Table 2a). Boiled and blanched tissue had similar amount of soluble sugars, as the water remained after these thermal processes, while steamed tissue contained a level that was 80 times higher than its associated water. Water remaining after steaming kale had only 0.14 ± 0.06 mg SucE/g fw of soluble sugars, probably due to the fact that the tissue was not immersed into water and encapsulated most of the soluble sugars. Similar results were shown in air-fried and steamed broccoli, which had a high amount of soluble sugars compared to other cooking methods [19]. However, it was shown that stir-frying lowers the content of soluble sugars up to 17.42% in bamboo shoots [65]. The high levels of soluble sugars observed in air-fried kale may be attributed to the exposure of the vegetable to a hot, dry environment with circulating air, which promotes water loss and results in a crisp texture. Consequently, the sugar concentration per gram of kale increases. A similar phenomenon has been reported in orange-fleshed sweet potato, where roasting led to a higher measured sugar content [66].

The soluble sugar level of thermally processed chard was the highest in the water remaining after boiling (11.33 ± 5.68 mg SucE/g fw) (Table 2b), probably due to extraction into the water. Losses of soluble sugars in samples exposed to dry heat or oil could be due to the Maillard reaction, where oil reduces the soluble sugars, which corresponds to the theory of the Maillard reaction degrading soluble sugars [39]. There are no other reports on the soluble sugar levels in chard during thermal processing.

The increase in sugar release from kale and chard during cooking is primarily due to the breakdown of plant cell walls and the solubilization of intracellular carbohydrates. Heat treatment disrupts the pectin and cellulose matrix in the cell walls, making sugars stored in vacuoles and other compartments more accessible to the cooking medium [67]. In addition, thermal degradation of complex polysaccharides, such as starch or hemicellulose, can produce smaller soluble sugars that contribute to the measured sugar content in the cooking water [68]. Factors such as cooking temperature, time, and method (boiling versus steaming) influence the extent of cell wall breakdown and sugar solubilization [69]. Therefore, the observed increase in sugar release is a combination of physical cell wall disruption and chemical breakdown of carbohydrate polymers during thermal processing.

The hydrogen peroxide level was the highest in boiled (1.47 ± 0.38 mM/g fw) and air-fried kale (1.64 ± 0.19 mM/g fw) (Table 2a). These results correspond to air-fried broccoli, in which the highest amount of H_2_O_2_ was also detected compared to other cooking methods [19]. Hydrogen peroxide is often formed in cooked vegetables such as onion, leek, and broccoli, due to high temperatures [70]. Hydrogen peroxide is released from cells as a result of polysaccharide wall degradation, and because the extracellular matrix plays a key role in its formation, the production of H_2_O_2_ during high-temperature cooking methods is not uncommon [67]. Water that remained after cooking kale had a lower amount of H_2_O_2_ (0.91 ± 0.18 mM/g fw) than the associated tissue, while there was no significant difference between the tissue and the water remaining after blanching and steaming. More than 95% of peroxidase activity in sweetcorn (*Zea mays*) was shown to be lost in hot water after steaming and blanching due to peroxidase activity, depending on the cooking method and duration and temperature of cooking [71].

Air-fried chard contained the most H_2_O_2_ as well (14.96 ± 3.14 mM/g fw) (Table 2b). The level of H_2_O_2_ was higher in the water remaining after boiling (4.09 ± 2.38 mM/g fw), but lower in the water remaining after blanching and steaming (0.61 ± 0.11 mM/g fw and 0.37 ± 0.23 mM/g fw, respectively) than in their associated tissue, since these methods of cooking are less disruptive than boiling.

### 2.5. Influence of Thermal Processing Techniques on Chlorophyll, Carotenoids, and Porphyrins in Kale and Chard

Thermal processing affected the content of photosynthetic pigments in kale (Table 3a). The highest amount of porphyrins and chlorophyll *b* was recorded in pan-fried kale (92.54 ± 31.68 mg/kg fw and 18.91 ± 11.42 mg/kg fw, respectively). The level of carotenoids, β-carotene, and lycopene was the highest in boiled kale (9.91 ± 3.71 mg/kg fw, 0.99 ± 0.32 mg/kg fw and 1.48 ± 0.42 mg/kg fw, respectively), while both pan-fried and boiled kale contained the most chlorophyll *a* (19.96 ± 3.37 mg/kg fw and 21.95 ± 5.67 mg/kg fw, respectively). On the other hand, thermal processing did not significantly change the content of any photosynthetic pigments in chard (Table 3b). Contrary to our results, Akdaş et al. [32] showed that stir-fried kale had fewer total chlorophylls and carotenoids than boiled and steamed. Boiling and stir-frying resulted in a decrease in chlorophyll content in broccoli heads [21,72]. However, our previous results on broccoli heads showed that chlorophyll *a*, chlorophyll *b*, porphyrins, and β-carotene did not significantly differ between samples from different cooking techniques [19]. Similarly to our results, the chlorophyll content did not differ between boiled and steamed samples of broccoli [72]. Air-fried broccoli had the highest concentration of carotenoids and lycopene, whereas pan-fried broccoli had the lowest content of carotenoids [19]. Although carotenoids are sensitive to the high temperatures of oil or hot air [73], our results showed that boiled kale retained the highest carotenoid concentration. This may be attributed to the rigid cell walls and membranes of kale. Carotenoids are primarily embedded in chromoplast membranes [74], and thermal processing can enhance their accessibility to solvents. Unlike high-temperature dry methods, such as pan-frying or air-frying, boiling reduces the formation of free radicals due to the lower temperature of the water [39]. High temperature can cause oil to produce hydroperoxide radicals, which leads to the degradation of carotenoids [73]. Yuan et al. [21] recorded a lower content of carotenoids in boiled broccoli than steamed and stir-fried, which is contrary to our results. Boiled cauliflower also had the lowest carotenoid content [25]. Boiled broccoli also had the lowest concentration of lycopene [19], which is usually degraded during high temperatures [75]; however, boiled kale had the highest concentration (Table 3a).

Chard showed no significant differences in photosynthetic pigment levels across the various thermal processing methods (Table 3b). Chard has relatively robust cell walls, and because pigments are bound within the chromoplast cell walls, it is likely that the different cooking treatments did not sufficiently disrupt the extracellular matrix to release pigments from the plastids.

### 2.6. Chemometric Analysis

#### 2.6.1. Principal Component Analysis

**Figure 3:**
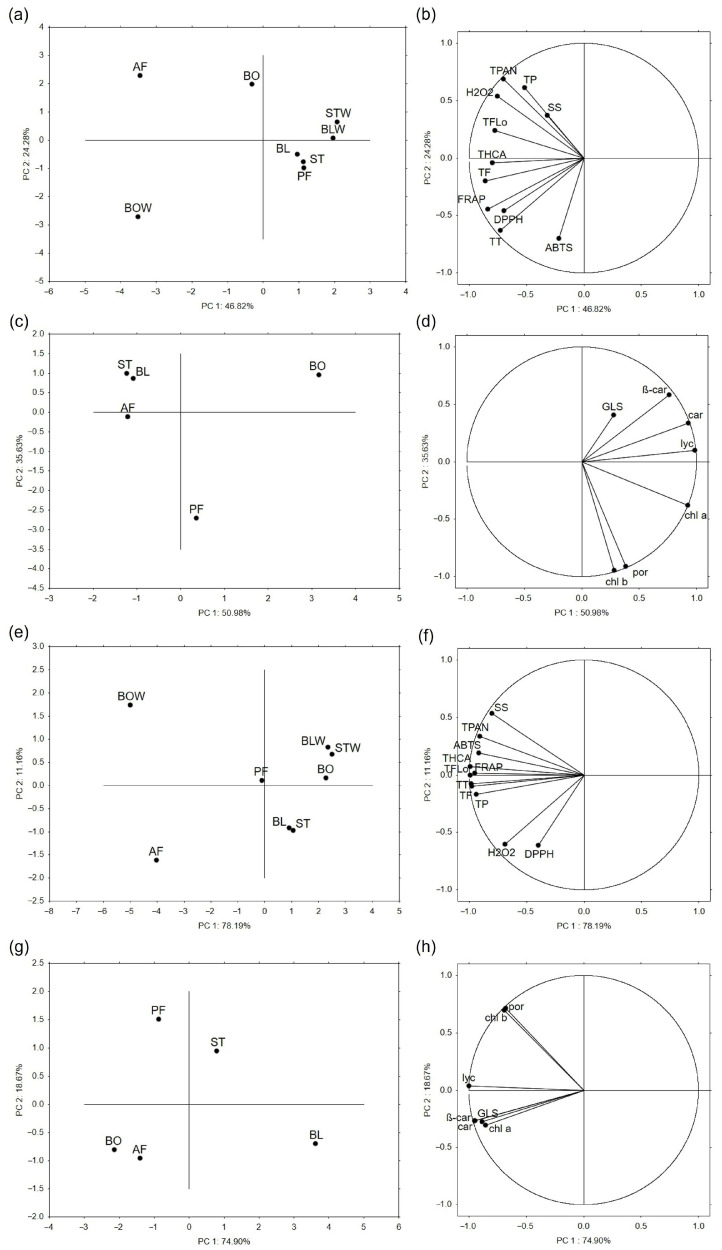
The principal component analysis shows the relationship between thermally processed (**a**,**c**) kale and (**e**,**g**) chard based on the analyzed variables, whose grouping is shown in the (**b**,**d**) for kale and (**f**,**h**) for chard parts of the figure. BO = boiled; BOW = water remaining after boiling; BL = blanched; BLW = water remaining after blanching; ST = steamed; STW = water remaining after steaming; PF = blanched and then pan-fried; AF = air-fried; SS = soluble sugars, TF = total flavonoids; TFlo = total flavonols; THCA = total hydroxycinnamic acids; TP = total phenolics; TPAN = total proanthocyanidins; TT = total tannins; β-car = β-carotene; car = carotenoids; *chl a* = chlorophyll *a*; chl *b* = chlorophyll *b*; GLS = total intact glucosinolates; lyc = lycopene; and por = porphyrins.

Principal component analysis (PCA) is used to reduce the number of dimensions (variables) in large data sets to two main PCs, which retain most of the original data, to increase interpretability and help recognize patterns in the data. Based on the antioxidant potential, phenolic compounds, soluble sugars, and H_2_O_2_ level of thermally processed kale samples, the first two PCs explained 71.1% of total variance (Table S1a). On the PCA plot, it is evident that steamed and blanched samples (tissues and remaining water) form a cluster with pan-fried kale, whereas samples are separated after boiling and air-frying (Figure 3a). The level of H_2_O_2_, total phenolics, flavonols, and proanthocyanidins contributed the most to the separation of the air-fried kale, while total tannins and the antioxidant potential measured by DPPH and FRAP contributed the most to the separation of water remaining after boiling. On the other hand, based on the glucosinolates and photosynthetic pigments of thermally processed kale, the first two PCs explained 86.6% of the total variance (Table S1b). As shown in Figure 3c, steamed, blanched, and air-fried kale formed a cluster, whereas boiled and pan-fried kale were separated. Variables that contributed the most to the separation of boiled kale were carotenoids, lycopene, and β-carotene, and to the separation of pan-fried kale, chlorophyll *b* and porphyrins.

Similar patterns to those for kale can be observed on the PCA plot of thermally processed chard based on their antioxidant potential, phenolic compounds, soluble sugars, and H_2_O_2_ level (Figure 3e). However, steamed and blanched chard samples were more similar to cooked than pan-fried chard. Again, the water remaining after boiling and air-fried chard was separated. Within the cluster, steamed and blanched tissue extracts were very similar, and a little more separate from the rest. The first two PCs explained 89.3% of the total variance (Table S1c). Soluble sugars, proanthocyanidins, and antioxidant potential measured by ABTS contributed most to the differentiation of the water remaining after boiling, whereas the H_2_O_2_ levels and antioxidant potential measured by DPPH were the main factors separating air-fried chard. Based on glucosinolate and photosynthetic pigment profiles (Figure 3g), boiled and air-fried chard clustered together, while the other samples were distinctly separated. The first two PCs explained 93.6% of the total variance (Table S1d). Variables that contributed the most to the separation of boiled and air-fried chard cluster were β-carotene, carotenoids, total intact glucosinolates, and chlorophyll *a*, while porphyrins and chlorophyll *b* contributed the most to the separation of pan-fried chard.

#### 2.6.2. Hierarchical Clustering

**Figure 4:**
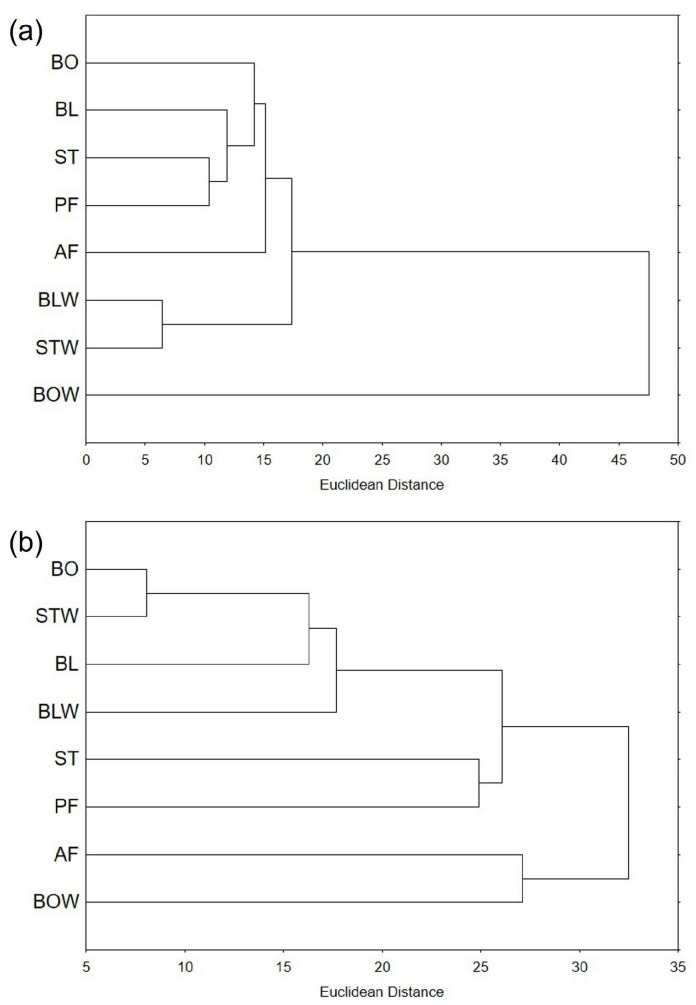
Hierarchical clustering of thermally processed (**a**) kale and (**b**) chard, expressed as Euclidean distance, based on the measured bioactive compounds and antioxidant potential. BO = boiled; BL = blanched; ST = steamed; PF = blanched and then pan-fried; AF = air-fried; BLW = water remaining after blanching; STW = water remaining after steaming; and BOW = water remaining after boiling.

Hierarchical clustering is used to identify groupings within a set of data, based on their similarity. Looking at dendrograms, it is easier to understand relationships between data points and gain insight into data structure. On the dendrogram based on the measured total bioactive compounds and antioxidant potential (Figure 4a), the water remaining after boiling kale is the most distant from all other samples, which form a cluster, VI. Water remaining after steaming and blanching was the most similar, and this cluster, I, was separated from the cluster V, containing all tissue extracts. Among the tissue samples, air-fried kale was the most distant, and steamed and pan-fried kale were the most similar.

Thermally processed chard samples were more similar to each other than kale samples, with a Euclidean distance of approximately 32 for chard and 48 for kale (Figure 4). However, when the water remaining after boiling kale was excluded, the remaining kale samples were much more similar, with a Euclidean distance of up to 18. The most similar kale samples were also more alike than the most similar chard samples—the Euclidean distance between the water remaining after blanching and steaming kale was approximately six, whereas the distance between boiled chard and the water remaining after steaming was eight.

Regarding thermally processed chard, the water remaining after boiling and air-frying chard formed a separate cluster, VI, from the other samples, but they were relatively distant from each other (around 27 Euclidean distance) (Figure 4b). In the inner cluster, similar to kale, steamed and pan-fried chard formed a cluster, IV, but were much more distant in chard than in kale (Euclidean distances 25 and 10, respectively). Boiled chard and the water remaining after steaming were the most similar.

#### 2.6.3. Pearson’s Correlation Analysis

Pearson’s correlation coefficient indicates the strength and direction of the linear relationship between two parameters. Correlation coefficients between measured variables in thermally processed kale and chard are shown in Table S2. According to Evans [76], the antioxidant potential measured by DPPH was very strongly positively correlated with the antioxidant potential measured by FRAP and the amount of soluble sugars (*r* = 0.895 and *r* = 0.887, respectively), while the antioxidant potential measured by FRAP was also very strongly positively correlated with the amount of soluble sugars (*r* = 0.924) and additionally to the amount of total tannins (*r* = 0.953). A very strong positive correlation was also observed between the total phenols and total flavonoids, proanthocyanidins and the H_2_O_2_ level (*r* = 0.986, *r* = 0,966, and *r* = 0.993, respectively), and total flavonoids and proanthocyanidins, H_2_O_2_ level, and chlorophyll *b* (*r* = 0.913, *r* = 0,971, and *r* = 0.815, respectively). Proanthocynidins were also very strongly positively correlated with the H_2_O_2_ level (*r* = 0.960). A very strong negative correlation has been recognized between the total flavonols and glucosinolates (*r* = −0.959) and the β-carotene and antioxidant potential measured by DPPH and FRAP, total tannins, and soluble sugars (*r* = −0.968, *r* = −0.921, *r* = −0.830, and *r* = −0.817, respectively). All photosynthetic pigments were very strongly positively correlated with each other, except for the correlation between chlorophyll *b* and β-carotene, which was strong (*r* = 0.788).

For thermally processed chard, the antioxidant potential measured by ABTS was strongly positively correlated with the antioxidant potential measured by FRAP, and FRAP values were strongly correlated with the total phenol content. Both ABTS and FRAP were also strongly positively correlated with total flavonols, tannins, hydroxycinnamic acids, soluble sugars, and H_2_O_2_ levels. Additionally, all measured phenolic compounds were strongly positively correlated with soluble sugars and H_2_O_2_. A very strong positive correlation was also observed between glucosinolates and carotenoids, *β*-carotene, and lycopene (*r* = 0.856, *r* = 0.976, and *r* = 0.872, respectively). A very strong positive correlation has also been recognized between the amount of chlorophyll *a* and carotenoids, β-carotene and lycopene (*r* = 0.946, *r* = 0.88, and *r* = 0.838, respectively), chlorophyll *b* and porphyrins (*r* = 0.946), carotenoids and β-carotene and lycopene (*r* = 0.946 and *r* = 0.941, respectively), and β-carotene and lycopene (*r* = 0.936).

## 3. Materials and Methods

### 3.1. Vegetable Samples

Mature kale (*Brassica oleracea* var. *acephala*) and chard (*Beta vulgaris* L. var *cycla*) were purchased in the Konzum store in Zagreb, Croatia (autumn 2024). The plant materials used in this study (kale and chard) were purchased from a commercial supermarket, rather than collected from the wild or a research collection. As such, no herbarium voucher specimens were prepared. The materials were identified based on the labeling provided by the supplier, which ensures that they correspond to the common market varieties of kale and chard. Outer kale leaves and chard leaf blades were processed using five cooking methods, homogenized, and extracted in 70% ethanol (general analyses) or 80% ethanol (pigments and intact glucosinolates) to a final concentration of 300 mg/mL. Cooking water from boiling, blanching, and steaming was collected, filtered (Whatman Grade 1), and analyzed alongside untreated tissues. Three biological and four technical replicates were prepared for each sample.

### 3.2. Applied Cooking Techniques of Kale and Chard

Five thermal processing techniques were applied—boiling (BO), blanching (BL), steaming (ST), pan-frying after blanching (PF), and air-frying (AF)—and selected as common methods for cruciferous vegetables [19]. These cooking methods are used worldwide; however, we modified the cooking duration and temperature. Conditions were adapted from the literature to reflect typical Croatian culinary practices. Boiling was performed at 100 °C for 30 min, blanching for 30 s, and steaming for 3 min, with all treatments conducted at a 300 mg/mL vegetable concentration. The remaining water from these methods (BOW, BLW, STW) was filtered for analysis. Blanched kale and chard were pan-fried for 5 min in sunflower oil (10:1 vegetable-to-oil ratio). Air-frying was carried out at 200 °C for 4 min in an air frier EAF7SB (Electrolux d.o.o., Zagreb, Croatia). All aqueous extracts and processed tissues were stored at −20 °C for further analysis.

### 3.3. Plasmid DNA Extraction

Plasmid pSgM1_HNF1A (gift from Prof. Dr. V. Zoldoš) was isolated from NEB^®^ Stable Competent *E. coli* using the NucleoBond Xtra Maxi kit (Macherey-Nagel, Düren, Germany), a standard transfection-grade plasmid purification method based on affinity column binding [19,77,78]. The plasmid carries a spectinomycin resistance cassette, enabling selection on LB plates.

### 3.4. DNA Nicking Protection Assay Using Fenton’s Reagent

A DNA nicking protection assay was performed using the pSgM1_HNF1A plasmid (400 ng/µL, 2854 bp), incubated with 70% ethanol or water extracts (300 mg/mL) and Fenton’s reagent, following Andonova et al. [76]. Reactions were carried out at 37 °C for 30 min. Trolox (30 mg/mL) served as the positive control, and plasmid with Fenton’s reagent as the negative control. Samples were separated on 1.5% agarose gels in TBE buffer for 2.5 h at 80 V, stained with GelRed^®^, and analyzed using ImageJ (v1.54g). A standard curve of pixel density versus DNA mass was used to quantify the DNA in each sample.

### 3.5. Determination of Antioxidant Capacity

The antioxidant capacity of the extracts was assessed using a Fluostar Optima microplate reader (BMG LABTECH, Ortenberg, Germany), employing down-sized ABTS, DPPH, and FRAP assays [79]. Calibration curves were constructed with Trolox standards (0.09 to 3.13 mg/mL). Results are expressed as percentage inhibition for the ABTS and DPPH assays, and as percentage reduction for the FRAP assay, calculated according to the following equations:

(1) $$\%\;\mathrm{inhibition}\;=\;\frac{{Abs}_{0}-{Abs}_{t}}{{Abs}_{0}}\;\times 100$$

(2) $$\%\;\mathrm{reduction}=\frac{{Abs}_{t}-{Abs}_{0}}{{Abs}_{t}}\;\times 100$$

where Abs_t_ = absorbance of the extract and Abs_0_ = absorbance of control (water or 70% ethanol).

### 3.6. Quantification of Polyphenolic Bioactive Compounds and Total Intact Glucosinolates

Polyphenolic compounds were quantified in 70% ethanol tissue extracts and in aqueous cooking-water extracts, all prepared at 300 mg fw/mL. Absorbance was measured using a Fluostar Optima microplate reader.

Total phenolics were determined by the Folin–Ciocâlteu method [79] at 740 nm, using gallic acid (0.02–1.25 mg/mL) for calibration and expressed as mg GAE/g fw.

Total tannins were measured following Galvão et al. [80] at 740 nm, also expressed as mg GAE/g fw.

Proanthocyanidins were quantified according to Weidner et al. [81] at 485 nm, using catechin (0.47–7.50 mg/mL) as a standard, expressed as mg CatE/g fw.

Flavonoids were determined via the AlCl_3_ colorimetric assay [82] at 520 nm, calibrated with quercetin (0.08–1.25 mg/mL) and expressed as mg QE/g fw.

Flavonols and hydroxycinnamic acids were measured as previously described [79] at 355 nm and 320 nm, using quercetin (0.02–0.31 mg/mL) and caffeic acid (0.08–1.25 mg/mL) standards, respectively.

Total intact glucosinolates were extracted in 80% acetone (300 mg/mL) and quantified as described in [50] at 425 nm using sinigrin (0.30–1.00 mg/mL) for calibration, expressed as mg SinE/g fw.

### 3.7. Measurement of Soluble Sugars and Hydrogen Peroxide Content

Soluble sugars were quantified using the sulfuric acid colorimetric method of Dubois et al. [51], with absorbance measured at 485 nm and sucrose standards (0.06–1.00 mg/mL) on a Fluostar Optima microplate reader. Results were expressed as mg sucrose equivalents per gram of fresh weight (mg SucE/g fw).

Hydrogen peroxide (H_2_O_2_) was measured following Junglee et al. [83] at 405 nm, using H_2_O_2_ standards (0.12–1.50 mM), and concentrations were expressed as mM H_2_O_2_/g fw.

### 3.8. Analysis of Photosynthetic Pigments (Chlorophyll and Carotenoids) and Porphyrins

Photosynthetic pigments were quantified following Sumanta et al. [84]. Extracts for chlorophylls, carotenoids, and porphyrins were prepared in 80% acetone at 300 mg/mL. Absorbance was recorded at 470, 575, 590, 628, 647, and 663 nm, using a Nanodrop 2000c (Thermo Fisher Scientific Inc., Waltham, MA, USA). Chlorophyll *a* and *b*, carotenoids, porphyrins, β-carotene, and lycopene were calculated using the equations provided in Table S3 [85].

### 3.9. Statistical Analysis

Data were analyzed in Statistica 14.1.0.8 (TIBCO Software Inc., Palo Alto, CA, USA). One-way ANOVA followed by Duncan’s multiple range test (*p* ≤ 0.05) was used to assess differences among groups, based on three biological and four technical replicates. PCA was performed to evaluate relationships among samples, and hierarchical clustering (HC) using Euclidean distance was used to examine sample grouping. Pearson correlation coefficients were calculated to assess parameter relationships, with values of 0.80–1.00 being considered very high [76].

## 4. Conclusions

Thermal processing strongly affects the phytochemical profile and bioactivity of vegetables. This study compared boiling, blanching, steaming, pan-frying, and air-frying on kale and chard, including their cooking waters. Chard generally showed stronger DNA-protective effects, with steamed chard and its water offering complete preservation; pan-fried kale was the most effective kale treatment. Boiling water from both vegetables has retained high antioxidant activity, underscoring the value of typically discarded cooking water. Steamed and air-fried tissues showed higher hydrophobic antioxidant capacity, while air-frying and cooking water preserved the most phenolics. Air-fried samples had the highest H_2_O_2_ levels, and chard contained more pigments, which were better protected due to its softer tissues; pan-frying best preserved pigments overall. Multivariate analyses revealed similarities between air-fried tissues and cooking waters and distinct differences between kale tissues and their water. These findings highlight the need to optimize processing to retain bioactive compounds and to valorize nutrient-rich cooking water as a sustainable by-product for food or nutraceutical applications.

## Figures and Tables

**Table 1:** Effect of different thermal processing techniques on different groups of polyphenolics and total intact glucosinolates in (**a**) kale and (**b**) chard.

**(a)**	**TP** **(mg GAE/g fw)**	**TT** **(mg GAE/g fw)**	**TPAN** **(mg CatE/g fw)**	**TF** **(mg QE/g fw)**	**TFlo** **(mg QE/g fw)**	**THCA** **(mg CAE/g fw)**	**GLS** **(mg SinE/g fw)**
BO	0.43 ± 0.24 a	0.09 ± 0.02 bcd	0.09 ± 0.03 b	0.52 ± 0.10 b	0.16 ± 0.08 c	1.56 ± 0.60 b	1.21 ± 0.72 a
BOW	0.35 ± 0.06 abc	0.25 ± 0.09 a	0.06 ± 0.02 c	0.99 ± 0.28 a	0.29 ± 0.05 b	2.20 ± 0.36 a	
BL	0.15 ± 0.08 c	0.12 ± 0.09 bcd	0.02 ± 0.02 de	0.10 ± 0.04 c	0.30 ± 0.15 b	1.53 ± 0.37 b	1.25 ± 0.91 a
BLW	0.31 ± 0.13 abc	0.07 ± 0.03 cd	0.04 ± 0.02 cde	0.17 ± 0.05 c	0.16 ± 0.07 c	0.51 ± 0.26 c	
ST	0.20 ± 0.12 bc	0.11 ± 0.04 bcd	0.03 ± 0.02 cde	0.22 ± 0.11 c	0.04 ± 0.02 c	0.25 ± 0.18 c	1.30 ± 0.84 a
STW	0.39 ± 0.28 ab	0.05 ± 0.02 d	0.05 ± 0.01 cd	0.23 ± 0.14 c	0.07 ± 0.06 c	0.68 ± 0.52 c	
PF	0.16 ± 0.08 c	0.16 ± 0.05 b	0.02 ± 0.01 e	0.16 ± 0.02 c	0.12 ± 0.11 c	0.51 ± 0.30 c	1.22 ± 0.88 a
AF	0.48 ± 0.17 a	0.14 ± 0.05 bc	0.15 ± 0.05 a	0.51 ± 0.15 b	0.50 ± 0.04 a	1.56 ± 0.31 b	1.14 ± 0.82 a
**(b)**	**TP** **(mg GAE/g fw)**	**TT** **(mg GAE/g fw)**	**TPAN** **(mg CatE/g fw)**	**TF** **(mg QE/g fw)**	**TFlo** **(mg QE/g fw)**	**THCA** **(mg CAE/g fw)**	**GLS** **(mg SinE/g fw)**
BO	0.26 ± 0.12 c	0.10 ± 0.06 bcd	0.05 ± 0.02 cd	0.38 ± 0.08 c	0.22 ± 0.16 c	0.92 ± 0.41 de	1.41 ± 1.03 a
BOW	0.80 ± 0.06 a	0.43 ± 0.17 a	0.46 ± 0.22 a	1.74 ± 0.28 a	1.57 ± 0.07 a	7.83 ± 0.35 a	
BL	0.52 ± 0.16 b	0.19 ± 0.09 b	0.10 ± 0.01 cd	0.76 ± 0.37 b	0.29 ± 0.22 bc	1.81 ± 1.00 cd	1.14 ± 0.84 a
BLW	0.19 ± 0.11 c	0.03 ± 0.03 d	0.15 ± 0.11 c	0.19 ± 0.06 c	0.09 ± 0.06 c	0.37 ± 0.33 e	
ST	0.32 ± 0.13 c	0.17 ± 0.09 bc	0.03 ± 0.01 d	0.36 ± 0.03 c	0.33 ± 0.16 bc	0.94 ± 0.58 de	1.17 ± 0.94 a
STW	0.34 ± 0.18 c	0.07 ± 0.04 cd	0.05 ± 0.02 cd	0.34 ± 0.11 c	0.14 ± 0.09 c	0.54 ± 0.35 de	
PF	0.57 ± 0.23 b	0.18 ± 0.07 bc	0.12 ± 0.03 cd	0.81 ± 0.12 b	0.52 ± 0.27 b	2.66 ± 0.58 c	1.36 ± 1.01 a
AF	0.88 ± 0.21 a	0.37 ± 0.17 a	0.29 ± 0.15 b	1.86 ± 0.14 a	1.49 ± 0.60 a	6.49 ± 2.39 b	1.49 ± 1.08 a

Values represent average ± standard deviation of three biological and four technical replicates. Different letters indicate a significant difference among the values in a column (one-way ANOVA, Duncan’s test, *p* ≤ 0.05). TP = total phenolics; GAE = gallic acid equivalent; fw = fresh weight; TT = total tannins; TPAN = total proanthocyanidins; CatE = catechin equivalent; TF = total flavonoids; QE = quercetin equivalent; TFlo = total flavonols; THCA = total hydroxycinnamic acids; CAE = caffeic acid equivalent; GLS = total intact glucosinolates; SinE = sinigrin equivalent; BO = boiled; BOW = water remaining after boiling; BL = blanched; BLW = water remaining after blanching; ST = steamed; STW = water remaining after steaming; PF = blanched and then pan-fried; and AF = air-fried.

**Table 2:** Effect of different thermal processing techniques on soluble sugars and hydrogen peroxide level in (**a**) kale and (**b**) chard.

(a)	SS (mg SucE/g fw)	H_2_O_2_ (mM/g fw)	(b)	SS (mg SucE/g fw)	H_2_O_2_ (mM/g fw)
BO	2.50 ± 0.28 de	1.47 ± 0.38 a	BO	0.91 ± 0.57 b	2.11 ± 1.02 d
BOW	0.72 ± 0.62 ef	0.91 ± 0.18 b	BOW	11.33 ± 5.68 a	4.09 ± 2.38 c
BL	3.02 ± 0.61 d	0.37 ± 0.15 c	BL	0.93 ± 0.38 b	3.58 ± 1.24 c
BLW	3.51 ± 0.27 d	0.28 ± 0.12 c	BLW	1.25 ± 0.36 b	0.61 ± 0.11 e
ST	10.95 ± 4.71 b	0.39 ± 0.10 c	ST	1.09 ± 0.79 b	3.82 ± 0.61 c
STW	0.14 ± 0.06 f	0.41 ± 0.21 c	STW	0.33 ± 0.23 b	0.37 ± 0.23 e
PF	5.98 ± 1.60 c	0.74 ± 0.30 b	PF	1.86 ± 1.31 b	7.45 ± 1.46 b
AF	15.30 ± 0.83 a	1.64 ± 0.19 a	AF	2.67 ± 1.47 b	14.96 ± 3.14 a

Values represent average ± standard deviation of three biological and four technical replicates. Different letters indicate a significant difference among the values in a column (one-way ANOVA, Duncan’s test, *p* ≤ 0.05). SS = soluble sugars; SucE = sucrose equivalent; fw = fresh weight; BO = boiled; BOW = water remaining after boiling; BL = blanched; BLW = water remaining after blanching; ST = steamed; STW = water remaining after steaming; PF = blanched and then pan-fried; AF = air-fried.

**Table 3:** Effect of different thermal processing techniques on photosynthetic pigments, expressed as mg/kg fresh weight, in (**a**) kale and (**b**) chard.

**(a)**	**Porphyrins**	**Chlorophyll *a***	**Chlorophyll *b***	**Carotenoids**	**β-Carotene**	**Lycopene**
BO	34.83 ± 9.02 b	21.95 ± 5.67 a	4.63 ± 1.04 ab	9.91 ± 3.71 a	0.99 ± 0.32 a	1.48 ± 0.42 a
BL	11.91 ± 2.67 b	2.03 ± 0.75 b	1.36 ± 0.14 ab	0.53 ± 0.05 b	0.46 ± 0.41 ab	0.12 ± 0.03 b
ST	6.09 ± 1.74 b	1.64 ± 0.26 b	0.34 ± 0.23 b	1.11 ± 0.64 b	0.14 ± 0.07 b	0.20 ± 0.10 b
PF	92.54 ± 31.68 a	16.96 ± 3.37 a	18.91 ± 11.42 a	0.86 ± 0.00 b	nd	0.49 ± 0.16 b
AF	17.00 ± 7.87 b	5.50 ± 0.95 b	2.70 ± 0.67 ab	1.21 ± 0.37 b	0.16 ± 0.01 b	0.27 ± 0.04 b
**(b)**	**Porphyrins**	**Chlorophyll *a***	**Chlorophyll *b***	**Carotenoids**	**β-Carotene**	**Lycopene**
BO	76.43 ± 34.07 a	56.84 ± 23.91 a	12.33 ± 6.25 a	22.65 ± 7.55 a	1.66 ± 0.15 a	2.86 ± 0.72 a
BL	40.53 ± 0.50 a	23.89 ± 6.38 a	5.68 ± 0.36 a	6.64 ± 0.40 a	0.71 ± 0.05 a	1.25 ± 0.12 a
ST	82.68 ± 16.73 a	40.14 ± 20.70 a	13.01 ± 0.86 a	12.05 ± 7.19 a	0.92 ± 0.90 a	2.05 ± 0.97 a
PF	111.69 ± 31.73 a	33.79 ± 0.18 a	14.68 ± 3.65 a	14.41 ± 1.12 a	1.34 ± 0.28 a	2.55 ± 0.04 a
AF	77.68 ± 15.98 a	45.90 ± 6.75 a	9.91 ± 1.73 a	18.54 ± 5.68 a	1.67 ± 0.33 a	2.63 ± 0.49 a

Values represent average ± standard deviation of three biological and three technical replicates. Different letters indicate a significant difference among the values in a column (one-way ANOVA, Duncan’s test, *p* ≤ 0.05). fw = fresh weight; BO = boiled; BL = blanched; ST = steamed; PF = blanched and then pan-fried; and AF = air-fried.

## Data Availability

The data that support the findings of this study are available from the corresponding author I.Š., upon request.

## Supplementary Materials

The following supporting information can be downloaded at: https://www.mdpi.com/article/10.3390/plants14243808/s1. Table S1a: Eigenvalues of correlation matrix and related statistics for active variables of thermally processed kale samples, based on their antioxidant potential, phenolic compounds, soluble sugars, and H_2_O_2_ level; Table S1b: Eigenvalues of correlation matrix and related statistics for active variables of thermally processed kale samples, based on their glucosinolates and photosynthetic pigments; Table S1c: Eigenvalues of correlation matrix and related statistics for active variables of thermally processed chard samples, based on their antioxidant potential, phenolic compounds, soluble sugars, and H_2_O_2_ level; Table S1d: Eigenvalues of correlation matrix and related statistics for active variables of thermally processed chard samples, based on their glucosinolates and photosynthetic pigments; Table S2a: Pearson’s correlation coefficients between measured variables in thermally processed kale; Table S2b: Pearson’s correlation coefficients between measured variables in thermally processed chard; Table S3: Equations used for calculation of chlorophyll *a*, chlorophyll *b*, carotenoids, β-carotene, and lycopene.

## Abbreviations

The following abbreviations are used in this manuscript:

ABTS	2,2′-azino-bis(3-ethylbenzothiazoline-6-sulfonic acid)
DPPH	Ferric reducing antioxidant power
FRAP	2,2-diphenyl-1-picrylhydrazy
ROS	Reactive oxygen species
PCA	Principal component analysis
BO	Boiled
BOW	Water remaining after boiling
BL	Blanched
BLW	Water remaining after blanching
ST	Steamed
STW	Water remaining after steaming
PF	Blanched and then pan-fried
AF	Air-fried
GAE	Gallic acid equivalents
fw	Fresh weight
QE	Quercetin equivalents
CatE	Catechin equivalents
CAE	Caffeic acid equivalents
TP	Total phenolics
TT	Total tannins
TPAN	Total proanthocyanidins
TF	Total flavonoids
TFlo	Total flavonols
THCA	Total hydroxycinnamic acids
SucE	Sucrose equivalents
SS	Soluble sugars
SinE	Sinigrin equivalents
β-car	β-carotene
car	Carotenoids
chl *a*	Chlorophyll *a*
chl *b*	Chlorophyll *b*
GLS	Total intact glucosinolates
lyc	Lycopene
por	Porphyrins
