# Supplementary material for: Thermal Processing Techniques Differentially Modulate Phytochemicals, Antioxidant Potential, and Genoprotective Effects of Kale (Brassica oleracea var. acephala) and Chard (Beta vulgaris L. var. cycla)
Source: Plants (Basel). 2025 Dec 14;14(24):3808. doi: 10.3390/plants14243808 (PMC12737114; doi:10.3390/plants14243808)
Supplement: Supplementary file 1 [file plants-14-03808-s001.zip › Table S1.pdf]

Table S1a Eigenvalues of correlation matrix, and related statistics for active variables of thermally processed kale samples based on their antioxidant potential, phenolic compounds, soluble sugars and H_2O_2 level.

Value number	Eigenvalue	% Total variance	Cumulative Eigenvalue	Cumulative %
1	5.150670	46.82427	5.15067	46.8243
2	2.670821	24.28019	7.82149	71.1045
3	1.851191	16.82901	9.67268	87.9335
4	0.742261	6.74783	10.41494	94.6813
5	0.417476	3.79524	10.83242	98.4765
6	0.150135	1.36487	10.98255	99.8414
7	0.017445	0.15859	11.00000	100.0000

Table S1b Eigenvalues of correlation matrix, and related statistics for active variables of thermally processed kale samples based on their glucosinolates and photosynthetic pigments.

Value number	Eigenvalue	% Total variance	Cumulative Eigenvalue	Cumulative %
1	3.568385	50.97693	3.568385	50.9769
2	2.494272	35.63246	6.062657	86.6094
3	0.835214	11.93162	6.897871	98.5410
4	0.102129	1.45899	7.000000	100.0000

Table S1c Eigenvalues of correlation matrix, and related statistics for active variables of thermally processed chard samples based on their antioxidant potential, phenolic compounds, soluble sugars and H_2O_2 level.

Value number	Eigenvalue	% Total variance	Cumulative Eigenvalue	Cumulative %
1	8.601155	78.19232	8.60116	78.1923
2	1.227324	11.15749	9.82848	89.3498
3	0.732167	6.65606	10.56065	96.0059
4	0.234069	2.12790	10.79472	98.1338
5	0.153624	1.39658	10.94834	99.5304
6	0.039238	0.35671	10.98758	99.8871
7	0.012423	0.11294	11.00000	100.0000

Table S1d Eigenvalues of correlation matrix, and related statistics for active variables of thermally processed chard samples based on their glucosinolates and photosynthetic pigments.

Value number	Eigenvalue	% Total variance	Cumulative Eigenvalue	Cumulative %
1	5.243339	74.90484	5.243339	74.9048
2	1.307098	18.67283	6.550437	93.5777
3	0.436524	6.23606	6.986961	99.8137
4	0.013039	0.18627	7.000000	100.0000
