# Supplementary material for: Thermal Processing Techniques Differentially Modulate Phytochemicals, Antioxidant Potential, and Genoprotective Effects of Kale (Brassica oleracea var. acephala) and Chard (Beta vulgaris L. var. cycla)
Source: Plants (Basel). 2025 Dec 14;14(24):3808. doi: 10.3390/plants14243808 (PMC12737114; doi:10.3390/plants14243808)
Supplement: Supplementary file 1 [file plants-14-03808-s001.zip › Table S2.pdf]

Table S2a Pearson's correlation coefficients between measured variables in thermally processed kale.

	**ABTS**	**DPPH**	**FRAP**	**TP**	**TT**	**TPAN**	**TF**	**TFLo**	**THCA**	**GLS**	**SS**	**H_2O_2**	**por**	**chl *a***	**chl *b***	**car**	**β-car**	**lyc**
ABTS	1.000																	
DPPH	0.715	1.000																
FRAP	0.327	0.895	1.000															
TP	-0.440	-0.124	0.112	1.000														
TT	0.068	0.737	0.953	0.098	1.000													
TPAN	-0.424	0.053	0.341	0.966	0.349	1.000												
TF	-0.380	-0.183	-0.005	0.986	-0.050	0.913	1.000											
TFLo	-0.601	0.129	0.559	0.466	0.748	0.647	0.314	1.000										
THCA	-0.992	-0.620	-0.204	0.483	0.055	0.496	0.406	0.698	1.000									
GLS	0.455	-0.279	-0.667	-0.623	-0.783	-0.796	-0.484	-0.959	-0.564	1.000								
SS	0.429	0.887	0.924	0.342	0.788	0.507	0.269	0.399	-0.315	-0.600	1.000							
H_2O_2	-0.543	-0.208	0.065	0.993	0.086	0.960	0.971	0.518	0.583	-0.645	0.265	1.000						
por	-0.679	-0.795	-0.642	0.679	-0.578	0.501	0.738	0.044	0.625	-0.055	-0.451	0.722	1.000					
chl *a*	-0.510	-0.766	-0.710	0.617	-0.704	0.405	0.708	-0.163	0.442	0.122	-0.467	0.641	0.976	1.000				
chl *b*	-0.736	-0.714	-0.495	0.782	-0.416	0.641	0.815	0.217	0.705	-0.240	-0.318	0.827	0.982	0.928	1.000			
car	-0.406	-0.769	-0.780	0.511	-0.800	0.279	0.623	-0.313	0.324	0.272	-0.526	0.529	0.934	0.988	0.859	1.000		
β-car	-0.605	-0.968	-0.921	0.246	-0.830	0.032	0.334	-0.252	0.505	0.337	-0.817	0.307	0.878	0.889	0.788	0.902	1.000	
lyc	-0.424	-0.761	-0.759	0.543	-0.776	0.315	0.651	-0.280	0.346	0.235	-0.503	0.561	0.944	0.993	0.876	0.999	0.895	1.000

TP = total phenolics; TT = total tannins; TPAN = total proanthocyanidins; TF = total flavonoids; TFlo = total flavonols; THCA = total hydroxycinnamic acids; GLS = total intact glucosinolates; SS = soluble sugars, por = porphyrins; chl *a* = chlorophyll *a*; chl *b* = chlorophyll *b*; car = carotenoids; β-car = β-carotene; lyc = lycopene.

Table S2b Pearson's correlation coefficients between measured variables in thermally processed chard.

	ABTS	DPPH	FRAP	TP	TT	TPAN	TF	TFLo	THCA	GLS	SS	H_2O_2	por	chl *a*	chl *b*	car	β-car	lyc
ABTS	1.000																	
DPPH	0.227	1.000																
FRAP	0.914	0.079	1.000															
TP	0.720	0.157	0.835	1.000														
TT	0.822	0.419	0.812	0.938	1.000													
TPAN	0.754	0.152	0.777	0.967	0.951	1.000												
TF	0.748	0.207	0.776	0.973	0.965	0.998	1.000											
TFLo	0.878	0.242	0.820	0.906	0.964	0.966	0.963	1.000										
THCA	0.812	0.166	0.818	0.960	0.961	0.996	0.993	0.984	1.000									
GLS	0.552	-0.407	0.422	0.471	0.451	0.638	0.589	0.666	0.650	1.000								
SS	0.907	-0.005	0.924	0.898	0.880	0.922	0.908	0.947	0.948	0.703	1.000							
H_2O_2	0.897	0.147	0.899	0.938	0.950	0.964	0.959	0.984	0.984	0.645	0.984	1.000						
por	0.480	-0.589	0.484	0.036	-0.031	0.044	-0.001	0.149	0.108	0.467	0.414	0.253	1.000					
chl *a*	0.182	-0.181	-0.165	-0.217	-0.057	0.029	-0.013	0.168	0.059	0.680	0.102	0.065	0.292	1.000				
chl *b*	0.293	-0.535	0.202	-0.285	-0.283	-0.237	-0.282	-0.088	-0.167	0.349	0.137	-0.019	0.936	0.456	1.000			
car	0.255	-0.411	-0.007	-0.030	0.034	0.197	0.144	0.286	0.218	0.856	0.287	0.221	0.429	0.946	0.500	1.000		
β-car	0.431	-0.455	0.252	0.270	0.273	0.465	0.411	0.515	0.480	0.976	0.547	0.479	0.482	0.808	0.436	0.946	1.000	
lyc	0.421	-0.525	0.223	0.051	0.074	0.231	0.173	0.336	0.271	0.872	0.432	0.324	0.704	0.838	0.717	0.941	0.936	1.000

TP = total phenolics; TT = total tannins; TPAN = total proanthocyanidins; TF = total flavonoids; TFlo = total flavonols; THCA = total hydroxycinnamic acids; GLS = total intact glucosinolates; SS = soluble sugars, por = porphyrins; chl *a* = chlorophyll *a*; chl *b* = chlorophyll *b*; car = carotenoids; β-car = β-carotene; lyc = lycopene.
