# Supplementary material for: Thermal Processing Techniques Differentially Modulate Phytochemicals, Antioxidant Potential, and Genoprotective Effects of Kale (Brassica oleracea var. acephala) and Chard (Beta vulgaris L. var. cycla)
Source: Plants (Basel). 2025 Dec 14;14(24):3808. doi: 10.3390/plants14243808 (PMC12737114; doi:10.3390/plants14243808)
Supplement: Supplementary file 1 [file plants-14-03808-s001.zip › Table S3.pdf]

Table S3 Equations used for calculation of chlorophyll *a*, chlorophyll *b*, carotenoids, ß-carotene and lycopene.

$$Chlorophyll\ a = 12.25\ Abs_{663}\ - 2.79\ Abs_{647}$$
$$Chlorophyll\ b = 21.50\ Abs_{645}\ - 5.10\ Abs_{663}$$
$$Carotenoids = \frac{(1000\ Abs_{470}\ - 1.82\ Chl\ a - 85.02\ Chl\ b)}{198}$$
$$Porphyrins = \frac{(12.25\ Abs_{663}\ - 2.55\ Abs_{647})}{892} + \frac{20.31\ Abs_{647} - 4.91\ \ Abs_{663}}{906}$$
$$+ (196.25\ Abs_{575} - 46.6\ Abs_{590} - 58.68\ Abs_{628})$$
$$+\ (61.81\ Abs_{590} - 23.77\ Abs_{575} - 3.55\ Abs_{628})$$
$$+\ (42.59\ Abs_{628} - 34.32\ Abs_{575} - 7.25\ Abs_{590})$$
$$\beta - Carotene = 0.216\ Abs_{663} - 1.22\ Abs_{647} - 0.304\ Abs_{505} +\ 0.452\ Abs_{453}$$
$$Lycopene = -0.0458\ Abs_{663} + 0.204\ Abs_{647} - 0.304\ Abs_{505} +\ 0.452\ Abs_{453}$$

where Abs = absorbance.
